# Light-controlled motility in prokaryotes and the problem of directional light perception

**DOI:** 10.1093/femsre/fux045

**Published:** 2017-10-18

**Authors:** Annegret Wilde, Conrad W. Mullineaux

**Affiliations:** 1 Institute of Biology III, University of Freiburg, 79104 Freiburg, Germany; 2 BIOSS Centre of Biological Signalling Studies, University of Freiburg, 79106 Freiburg, Germany; 3 School of Biological and Chemical Sciences, Queen Mary University of London, Mile End Road, London E1 4NS, UK

**Keywords:** cyanobacteria, motility, photoreceptors, phototrophic prokaryotes, phototaxis, signal transduction

## Abstract

The natural light environment is important to many prokaryotes. Most obviously, phototrophic prokaryotes need to acclimate their photosynthetic apparatus to the prevailing light conditions, and such acclimation is frequently complemented by motility to enable cells to relocate in search of more favorable illumination conditions. Non-phototrophic prokaryotes may also seek to avoid light at damaging intensities and wavelengths, and many prokaryotes with diverse lifestyles could potentially exploit light signals as a rich source of information about their surroundings and a cue for acclimation and behavior. Here we discuss our current understanding of the ways in which bacteria can perceive the intensity, wavelength and direction of illumination, and the signal transduction networks that link light perception to the control of motile behavior. We discuss the problems of light perception at the prokaryotic scale, and the challenge of directional light perception in small bacterial cells. We explain the peculiarities and the common features of light-controlled motility systems in prokaryotes as diverse as cyanobacteria, purple photosynthetic bacteria, chemoheterotrophic bacteria and haloarchaea.

## Teaser

The authors discuss our understanding of the ways in which prokaryotes can control their motility in response to the intensity, spectral quality and direction of illumination.

## INTRODUCTION TO LIGHT-CONTROLLED MOTILITY AND PHOTORECEPTORS

### Scope and organization of this review

The study of photobehavior gives a direct window into the sensory world of prokaryotes. How do our smallest and most ancient relatives perceive and respond to their environment? Prokaryotic systems for control of motility by light are diverse, linking a wide range of light-generated signals to control of several distinct motility systems. However, in addition to great diversity, there are also common elements that appear in different contexts and in very different organisms. After some introductory remarks and definitions, we first discuss the individual elements of light-dependent motility: the motility systems, signal transduction components and the light sensors. We then discuss examples of the different ways in which these elements have been combined to produce light-controlled motility responses in different specific groups of organisms: haloarchaea, purple photosynthetic bacteria, chemoheterotrophic bacteria and cyanobacteria. We close with some unanswered questions and challenges for the future. We hope that this review will stimulate further research in the area including synthetic biology applications and the search for behavioral responses to light in a wider range of prokaryotes.

### Physiological advantages of light-controlled motility

The ability to link light perception to control of motility is found in a very wide variety of prokaryotes, indicating that this ability must confer a range of physiological advantages (Häder 1987; Armitage and Hellingwerf 2003). Most directly, the light environment is crucial to phototrophs as their energy source. Phototrophic prokaryotes are extraordinarily diverse, with a likely role for horizontal gene transfer in spreading phototrophy across multiple phyla (Raymond *et al.*^2002^). Thus, different groups of phototrophic prokaryotes may have little in common apart their exploitation of light as an energy source, but it should be advantageous for any phototroph to be able to relocate in search of better light environments for photosynthesis. To do this efficiently requires the ability to control motility in response to integrated information on the intensity of light, the spectral quality of light and the physiological status of the cell. A second major reason for light-controlled motility is to avoid light at damaging intensities or wavelengths: this factor is not confined to photosynthetic bacteria since light (especially in the UV region) can be dangerous to all prokaryotes, primarily because of DNA and protein damage (Rastogi *et al.*^2014^) and inhibition of the translation machinery by light-generated reactive oxygen species (Yutthanasirikul *et al.*^2016^). Finally, light signals potentially contain rich and complex information about the environment, and we should not exclude the possibility that bacteria make sophisticated use of this information to optimize their location and behavior. For example, plant or animal pathogens could use light information to control their location and interaction with their hosts, and in fact light signals are known to regulate development and virulence in several non-phototrophic prokaryotes (Purcell and Crosson 2008; Bonomi *et al.*^2016^). Phototrophs could also benefit from sophisticated information processing, since their optimal environment is defined by a complex combination of factors including light intensity, light quality, day and night cycles, the availability of raw materials and alternative energy sources, other beneficial or harmful physical and chemical factors and sometimes the presence of symbiotic partners. Light quality strongly influences specialized developmental pathways in certain filamentous cyanobacteria, including the development of motile hormogonia and nitrogen-fixing heterocysts (Damerval *et al.*^1991^). Since hormogonia are important for establishing symbiotic partnerships between cyanobacteria and plants, and heterocysts are essential for nitrogen fixation in those partnerships, it is tempting to speculate that the cyanobacteria may be using light signals as one way to detect the proximity of a plant symbiotic partner. Within a complex and heterogeneous environment such as a phototrophic biofilm, many factors crucial for growth could vary dramatically even within the limited region that a single motile cell could explore (Richardson and Castenholz 1987; Stal 1995). We should therefore expect that prokaryotes living in such environments might control their motility in response to a complex signal transduction network linking a range of environmental cues.

### Definitions of light-controlled motile behavior

**Figure 1.**
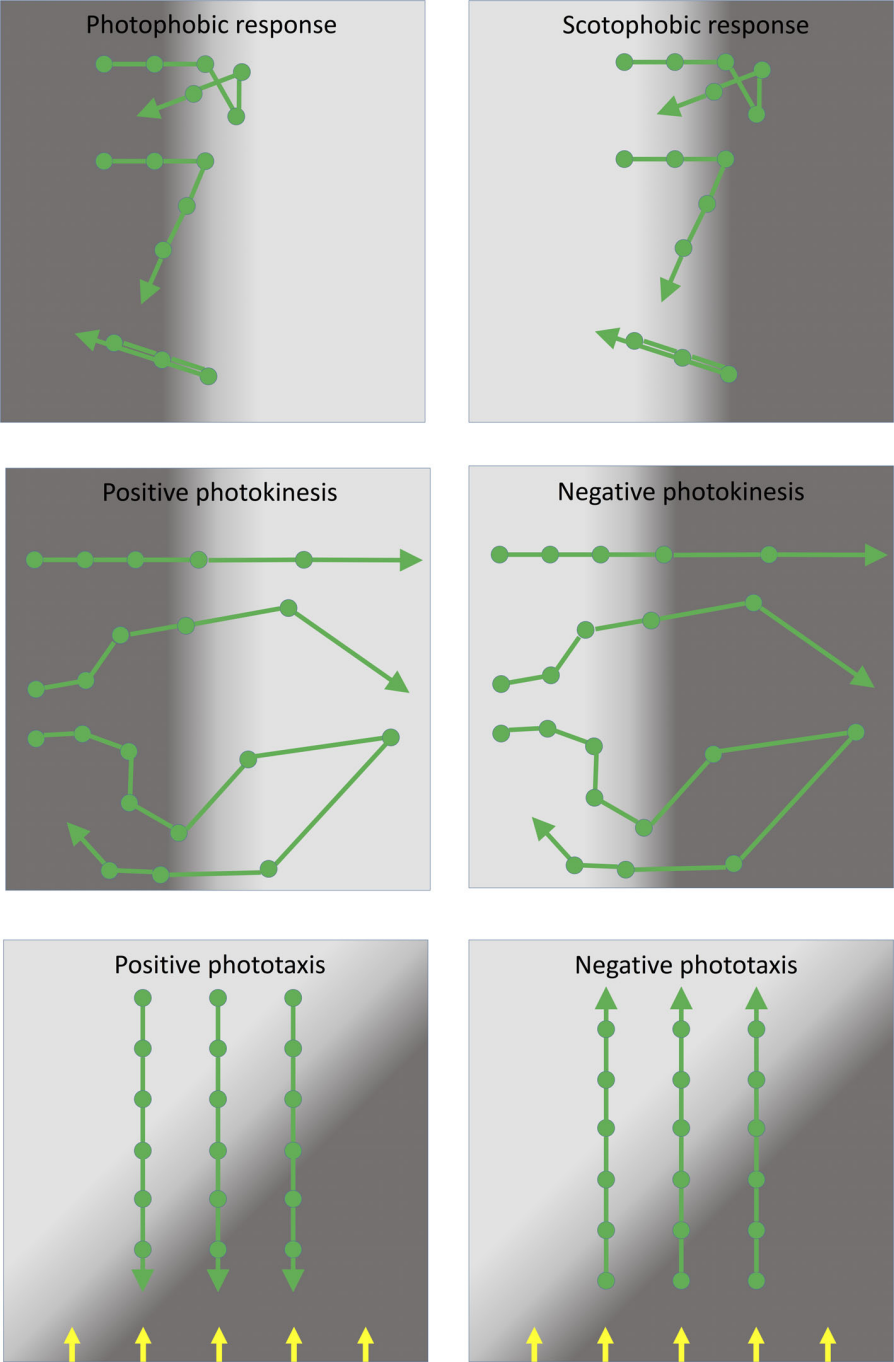
Cartoon to illustrate different types of photobehavior found in prokaryotes. Spaces between the filled circles represent equal time intervals. Top: photophobic and scotophobic responses involving random tumbling or 180° motility reversals induced by sudden changes in the light environment experienced by the cells. Middle: photokinesis involving changes in speed induced by changing light intensity. In patchy light environments, positive photokinesis results in accumulation in low light areas (and vice versa for negative photokinesis). Bottom: true phototaxis results in movement towards or away from a light source, but is not a response to a light gradient. Direction of parallel illumination is indicated by the yellow arrows.

As discussed below, prokaryotes utilize a rich variety of light-perception systems which may be linked to the control of several radically different motility mechanisms. However, regardless of the specific molecular mechanisms employed, it is useful to define several distinct forms of light-controlled behavior (Häder 1987). These definitions are used throughout the subsequent discussion, and the concepts are illustrated in schematic form in Fig. 1.

- The *photophobic response* is a change in the direction of motility in response to a relatively sudden increase in illumination: classically, the response is to a temporal change in light intensity, which the bacterium may experience as it moves into a brightly illuminated region. The directional switch may consist of a random selection of a new direction (‘tumbling’) or it may be a simple reversal in the direction of motility. Either has the effect of repelling cells from a patch of unfavorable light. Photophobic responses have been observed in prokaryotes as diverse as *Escherichia coli* (*E. coli*), purple photosynthetic bacteria and haloarchaea (Yang, Inokuchi and Adler 1995; Armitage and Hellingwerf 2003).
- The *scotophobic* (‘fear of darkness’) response is the converse of the photophobic response described above: a change in direction (tumbling or reversal) is induced when the cell experiences a relatively sudden drop in light intensity. Photophobic and scotophobic responses both cause cells to accumulate in regions of specific (presumably favorable) light intensity and spectral quality. Scotophobic responses have been well documented in purple photosynthetic bacteria, starting with the classic observations of Engelmann (1883), and in cyanobacteria (Häder 1987). Scotophobic/photophobic responses in flagellated bacteria closely resemble the classic ‘biased random walk’ mode of bacterial chemotaxis, which links perception of temporal changes in the concentration of a chemical attractant or repellent to the frequency of tumbling (Wadhams and Armitage 2004). The only significant distinction is that the scotophobic/photophobic responses involve perception of temporal changes in light intensity rather than the concentration of a chemical.
- *Photokinesis* is a light-induced change in the speed (but not direction) of movement. Photokinesis may be negative (light-induced reduction of motility) or positive (light-induced stimulation of motility). Photokinesis can cause cells to accumulate in regions of favorable illumination: they linger in such regions or accelerate out of regions of unfavorable illumination. Photokinesis has been documented in cyanobacteria and purple photosynthetic bacteria (Häder 1987).
- *True phototaxis* consists of directional movement which may be either towards a light source (positive phototaxis) or away from a light source (negative phototaxis). In contrast to the photophobic/scotophobic responses, true phototaxis is not a response to a temporal change in light intensity. Generally, it seems to involve direct sensing of the direction of illumination rather than a spatial gradient of light intensity. True phototaxis in prokaryotes is sometimes combined with *social motility*, which involves the concerted movement of an entire colony of cells towards or away from the light source. This phenomenon could also be described as *community phototaxis.* True phototaxis is widespread in eukaryotic green algae (Kreimer 2009), but among the prokaryotes it has been documented only in cyanobacteria (Häder 1987; Bhaya 2004), and in social motility of colonies of the purple photosynthetic bacterium *Rhodocista centenaria* (Ragatz *et al.*^1994^).

### Light perception in prokaryotes: direct versus indirect photosensing

**Figure 2.**
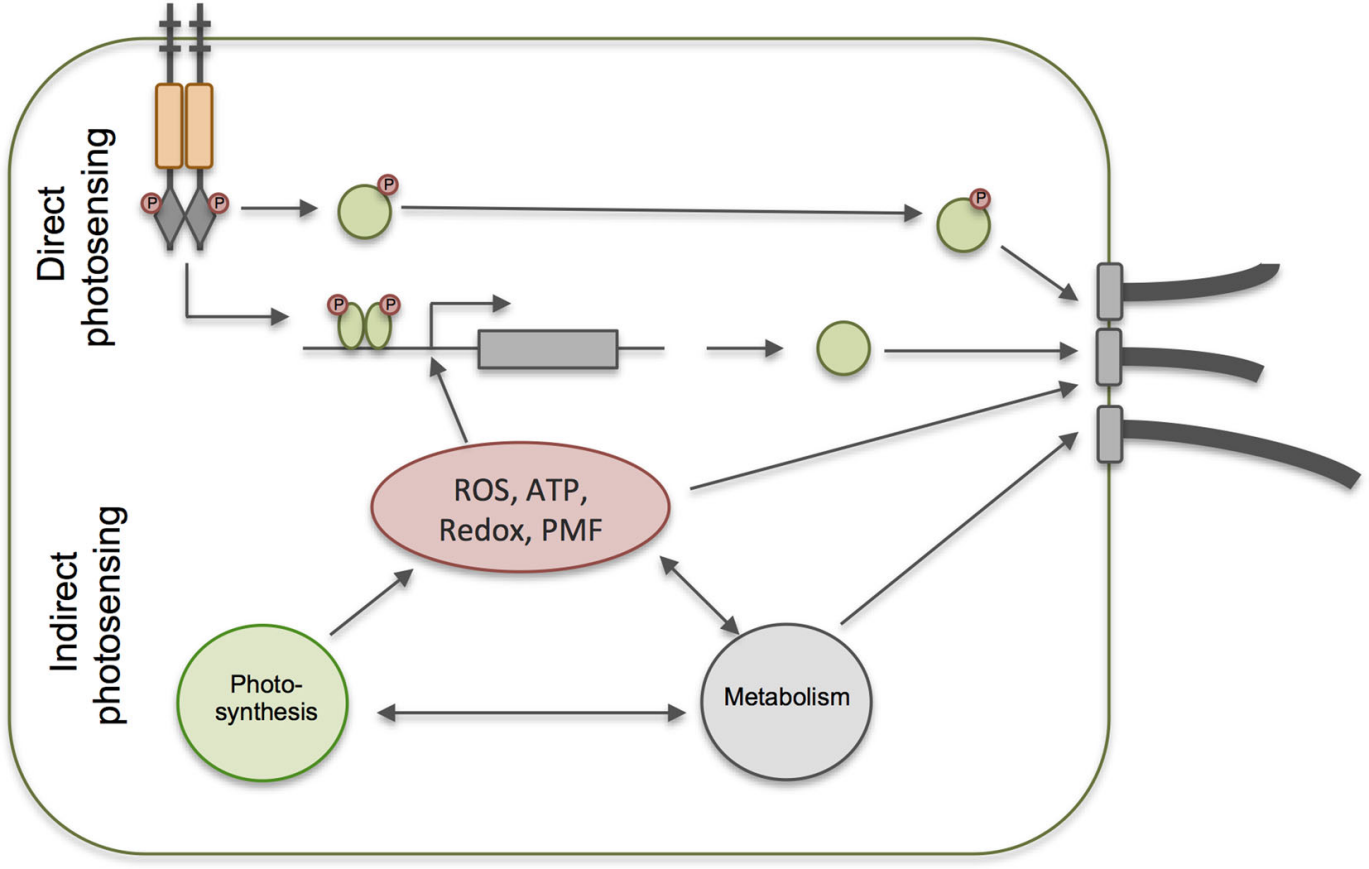
Direct versus indirect photosensing for control of photobehavior. Direct photosensing involves dedicated photoreceptor systems which trigger signal transduction for control of the motility apparatus and/or changes in gene expression leading to changes in photobehavior. With indirect photosensing, these processes are controlled in response to the products of photosynthesis (in phototrophs) or other by-products of illumination such as reactive oxygen species.

Direct photosensing employs dedicated photosensory chromophore-binding proteins (Table 1). These proteins change conformation upon absorption of a photon by the chromophore, triggering signal transduction which may eventually lead to responses including control of motility. Several families of proteins are involved in direct light perception and motility control in prokaryotes, as detailed below. In addition to the direct photosensors, it should be noted that light inputs can lead to signal transduction for motility control in a variety of indirect ways (Fig. 2). This is especially true for phototrophic prokaryotes, where light absorption by the photosynthetic apparatus generates a wealth of signals that could potentially control motility, in addition to most other activities in the cell. Such potential signals include light-generated transmembrane proton gradients, light-powered electron transport leading to changes in the redox state of electron carriers, and even more downstream effects such as changes in the ATP/ADP ratio and levels of metabolites and by-products. Although the potential for indirect photosensing is most obvious in phototrophic prokaryotes, there are also possible indirect photosensory signals in chemoheterotrophs. For example, light-induced production of reactive oxygen species has been suggested as a signal for a photophobic response in *E. coli* (Yang, Inokuchi and Adler 1995).

**Table 1.** Overview of prokaryotic photoreceptors.

Photoreceptor type	Wavelength(s) of light detected	Found in:	Physiological role
Sensory rhodopsin	Blue/orange (370/480/587 nm)	Haloarchaea	Photophobic/scotophobic responses^a^
	Green/orange (550–570 nm)	*Salinibacter ruber*	Possible role in phototaxis^b^
	Green/orange (480–590 nm)	Cyanobacteria (*Anabaena* sp.)	Regulation of phycobilisome composition^c^
Phytochrome	Red/far-red light	*Agrobacterium* sp.	Regulation of conjugation^d^
	(∼650/710 nm) or near infrared	*Pseudomonas* sp.	Regulation of motility and growth^e^
		Purple bacteria	Regulation of photosynthetic gene expression^f^
		Cyanobacteria	Unknown
Cyanobacteriochrome	UV-A to far-red, depending on the specific protein	Cyanobacteria	Positive phototaxis^g^Negative phototaxis^h^Regulation of motility/sessility^i^Cell aggregation^1^^j^Gene expression^h^Phycobilisome composition^k^
BLUF (sensor of blue light using FAD)	Blue light (380/440 nm)	CyanobacteriaPurple bacteria*E. coli*	Phototaxis^l^Photosynthetic gene expression^m^Regulation of motility and biofilm formation^n^
PYP (photoactive yellow protein)	Blue (450 nm)	Purple bacteria	Photophobic response in *Ectothiorhodospira halophila*^o^: function unknown in other purple bacteria
LOV (light, oxygen, voltage) domains	Blue (450 nm)	Cyanobacteria*Bacillus subtilis*	Regulation of c-di-GMP level^p^Possible trigger for chemotaxis^q^
Cryptochrome	UV/blue (380/442 nm)	Cyanobacteria	DNA repair activity^r^
			Possible role in negative phototaxis^s^
			Possible role in regulation of gene expression^s^
OCP (orange carotenoid-binding protein)	Blue-green (496 nm)	Cyanobacteria	Quenching of excess energy in phycobilisomes^t^

a Hoff, Jung and Spudich (1997)

b Kitajima-Ihara *et al.* (2008)

c Vogeley *et al.* (2004)

d Bai *et al.* (2016)

e Shah *et al.* (2016)

f ^f^Giraud *et al.* (2002)

g Bhaya, Takahashi and Grossman (2001)

h ^h^Song *et al.* (2011)

i Savakis *et al.* (2012)

j Enomoto *et al.* (2014)

k Hirose *et al.* (2008)

l Sugimoto *et al.* (2017)

m Gomelsky and Klug (2002)

n Tschowri, Busse and Hengge (2009)

o Sprenger *et al.* (1993)

p Cao *et al.* (2010)

q Gaidenko *et al.* (2006)

r Brudler *et al.* (2003)

s Moon *et al.* (2010a)

t Wilson *et al.* (2008)

Distinguishing direct and indirect photosensing is not always straightforward, given that in phototrophs, for example, light may be required to power motility in addition to being a potential trigger for control of motility. Therefore, inhibiting photosynthesis may not be a feasible approach to determining the role of the photosynthetic apparatus in controlling motility. Furthermore, there is always the potential for multiple information inputs from both direct and indirect photosensing. Action spectra for motility responses provide the most satisfactory way to demonstrate indirect photosensing by the photosynthetic apparatus: if that is the sole control for motility, then the action spectrum for the motility response should match the action spectrum for photosynthesis. Action spectra can also be helpful for demonstrating the roles of direct photoreceptors, but in cyanobacteria, for example, the most convincing demonstrations of the roles of specific photosensors have come from the altered motility phenotypes of specific knockout mutants (Bhaya *et al.*^2001^; Campbell *et al.*^2015^).

### Challenges of directional light perception in prokaryotes

The small size of prokaryotic cells presents some special challenges for perception of the surrounding environment. A classic instance occurs in chemotaxis, where unicellular bacteria appear too small for direct perception of chemical concentration gradients: most chemical gradients are not steep enough for the concentration of the chemical to be sufficiently different at the two ends of the cell, usually no more than a few microns apart. A rare exception occurs with gradients in O_2_, which apparently can be directly detected by a vibrioid bacterium (Thar and Kühl 2003). More typically, chemotactic bacteria such as *E. coli* rely on temporal rather than spatial perception of chemical gradients, for example, suppressing tumbling if the concentration of an attractant is increasing with time, or increasing the frequency of tumbling if the concentration decreases with time (Wadhams and Armitage 2004). An analogous problem occurs with gradients in light intensity, which in natural environments will rarely be steep enough for perceptible differences in light intensity across length scales of a few microns. The scotophobic and photophobic responses of prokaryotes conceptually resemble typical bacterial chemotaxis, in that they rely on the detection of temporal changes in the light environment. However, some unicellular bacteria do exhibit ‘true phototaxis’, which requires directional perception of light: the measurement not only of the intensity and spectral quality of a light source but also its position. This behavior has been characterized in unicellular cyanobacteria (Choi *et al.*^1999^; Schuergers *et al.*^2016^), filamentous cyanobacteria (Nultsch and Wenderoth 1983) and in swarming colonies of the purple photosynthetic bacterium *R. centenaria* (Ragatz *et al.*^1994^). With *R. centenaria* and the filamentous cyanobacteria, it is possible that intercellular communication and interactions may be involved in the mechanism of light-direction sensing. This would allow the cells to integrate information from a much larger area than that of a single cell. However, at least in the case of the unicellular cyanobacteria, it is clear that individual cells can sense light direction and control their motility accordingly. The unicellular cyanobacteria generally do exhibit social motility, moving in tightly packed colonies (Kondou *et al.*^2001^; Burriesci and Bhaya 2008). However, when cells are dispersed in a suitable environment so that they move independently, it is clear that the separated cells retain the ability to accurately detect the position of a light source and move towards it (Choi *et al.*^1999^; Schuergers *et al.*^2016^).

In pigmented cells, such as those of cyanobacteria, shading could in principle provide the basis for a mechanism of light-direction sensing: under directional illumination, a cell would have a bright illuminated side and a dark shaded side. A system linking light intensity perception to control of the motility apparatus would then respond differently to the distinct light intensities at the two sides of the cell, leading to differential activation/inactivation of the motility apparatus and thus directional motility. Such a model is more problematic than it appears, because individual cyanobacterial cells are too small to absorb a major proportion of the light passing through them. A direct measurement of the single cell absorption spectrum of *Nostoc* sp. indicates that about 10% of photons are absorbed at peak wavelengths (Sugiura and Itoh 2012), while an indirect estimate in phototactic cells of *Synechocystis* sp. PCC 6803 (hereafter *Synechocystis*) suggests that at most about 20% of photons are absorbed (Schuergers *et al.*^2016^). Shading-based directional motility would therefore require a system capable of making extremely sensitive comparisons between the light intensity and quality at the two sides of the cell. A solution to the problem was provided by a detailed study of light transmission through the spherical cells of *Synechocystis*, which showed that the cell acts as a very effective lens, focusing light at the edge of the cell *away* from the light source. This lensing effect is quantitatively much greater than the shading effect, and it provides the basis for directional light perception in *Synechocystis* (Schuergers *et al.*^2016^; Nakane and Nishizaka 2017). Interestingly, it now appears that the unicellular green alga *Chlamydomonas reinhardtii* uses a similar principle of lensing by the cell body, in conjunction with its ‘eyespot’ apparatus, for directional light perception (Ueki *et al.*^2016^). Microoptical lensing effects do not need to be confined to highly pigmented cells, opening up the possibility that some non-phototrophic prokaryotes could be capable of directional light perception.

## MOTILITY MECHANISMS IN PROKARYOTIC PHOTOMOVEMENT

Several fundamentally different motility mechanisms are involved in prokaryotic photobehavior (Table 1). Each system is briefly discussed, with particular regard to the ways in which motility can be regulated and the potential for the regulation to lead to different forms of light-controlled behavior.

### The flagellum

Flagella consist of helical polymeric fibers of flagellin subunits anchored to the flagellar motor, a complex that spans the cytoplasmic membrane and the periplasm. Swimming results from rotation of the flagella driven by a transmembrane gradient in H^+^ or Na^+^ and directional effects result from switches in the direction and/or speed of rotation (Wadhams and Armitage 2004). The number and location of flagella differ between species. *E. coli* has five to eight flagella distributed over the cell surface. Counterclockwise rotation causes the flagella to form a bundle whose rotation drives the cell forwards. A switch to clockwise rotation causes the flagellar bundle to fly apart, resulting in random tumbling rather than forward movement. *E. coli* swimming is therefore characterized by alternating phases of forward movement and random changes in direction, and chemotaxis in *E. coli* works by controlling the frequency of tumbling, in response to temporal changes in the external chemical environment (Wadhams and Armitage 2004). The chemical sensors and the flagellar motor are linked by a typical chemotaxis signal transduction system, as discussed below. Purple phototrophic bacteria have variable numbers of flagella. For example, *Rhodobacter sphaeroides* and *R. centenaria* normally have only a single flagellum, although this can vary in different growth conditions (Sackett *et al.*^1997^). By contrast, *Rhodospirillum rubrum* has tufts of flagella at one or both poles (Lee and Fitzsimons 1976). The single flagellum of *R. sphaeroides* does not undergo rotation reversals, rather it rotates counterclockwise to drive forward swimming (Sackett *et al.*^1997^). Reorientation of the cell occurs when the flagellum stops rotating, although the mechanism of reorientation is not understood. It had been assumed to be due to Brownian rotational diffusion of the cell, but some data suggest that an active reorientation mechanism is at work (Rosser *et al.*^2014^). Flagellated bacteria exhibit a range of photophobic and scotophobic responses, all dependent on the triggering by light signals of flagellar rotation reversal, pausing or changes in rotation speed (Romagnoli and Armitage 1999). In addition, there is a considerable older literature on photokinesis in flagellated purple bacteria (reviewed by Häder 1987) but it is not clear whether the photokinetic effects reflect genuine signal transduction in response to light signals or simply changes in the proton motive force due to changes in photosynthetic activity under different illumination.

### The type IV pilus

Type IV pili are multifunctional appendages found in many kinds of bacteria, including cyanobacteria (Schuergers and Wilde 2015). One of their functions is to enable a form of motility on surfaces, often referred to as ‘twitching’. Type IV pili are protein fibers composed of subunits of the major pilin, PilA. The type IV pilus machinery is well characterized in *Myxococcus xanthus* (Chang *et al.*^2016^). PilA subunits are hydrophobic and associated with the cytoplasmic membrane when the pilus is depolymerized. Pili are formed when the PilA subunits are exported from the cell via an inner membrane channel (PilC) and an outer membrane pore (PilQ). As they are translocated, the PilA subunits polymerize into an extended pilus. Pilus extension is powered by a cytoplasmic ATPase (PilB) that interacts with PilC at the cytoplasmic surface of the inner membrane. The tip of the pilus has an affinity for some surfaces, and, when the pilus tip binds to a surface, a signal seems to be transmitted back to the pilus base, resulting in the detachment of PilB and the binding of another motor ATPase, PilT (Chang *et al.*^2016^). PilT is an ATP-powered retraction motor that re-imports and disassembles the PilA subunits. It is the retraction of the surface-attached pilus that drags the cell body forward (Merz, So and Sheetz 2000). Type IV pili were initially characterized in chemoheterotrophs such as *Pseudomonas*, *Myxococcus* and *Neisseria*, but the system in cyanobacteria appears to work the same way, with conservation of all the major protein components (Schuergers *et al.*^2015^). As in other bacteria, motility in *Synechocystis* is driven by pilus retraction (Nakane and Nishizaka 2017).

**Figure 3.**
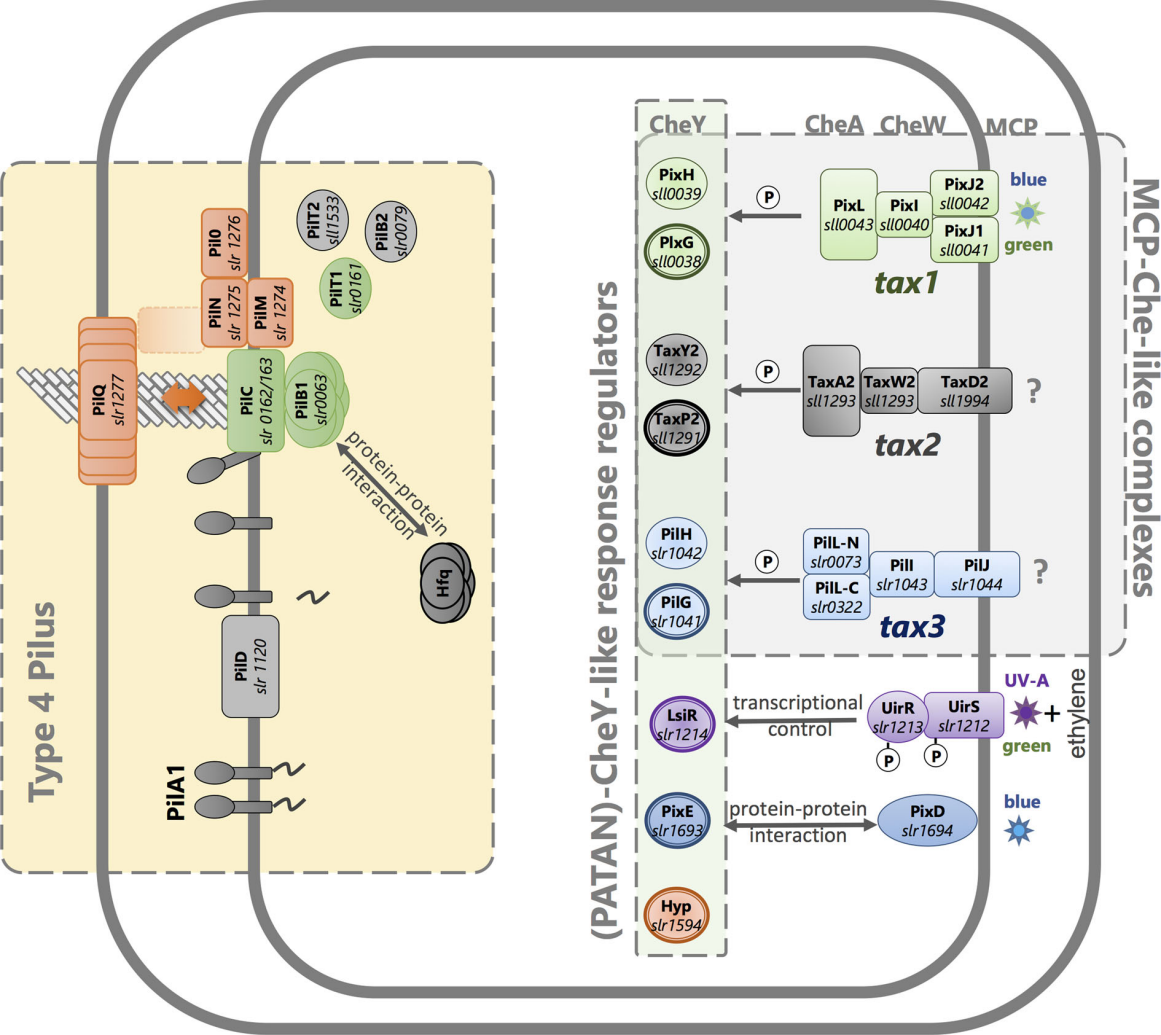
Motility control in the cyanobacterium *Synechocystis* systems for signal perception, signal transduction and motility. Known systems for photoperception are illustrated along with the products of the *tax2* and *tax3* operons, which are likely to control motility in response to uncharacterized stimuli. Bold outlines indicate CheY-like response regulators with PATAN domains. See Table 2 and text for further details and references.

In rod-shaped bacteria such as *Pseudomonas aeruginosa* and *M. xanthus*, the type IV pili are located close to the cell poles (Bulyha *et al.*^2009^; Burrows 2012). By contrast, in the spherical cyanobacterium *Synechocystis*, the type IV pili appear to be distributed over the entire cell surface, although sometimes asymmetrically grouped in clusters (Bhaya *et al.*^2000^). The parts of the type IV pilus machinery that span the cell surface layers and form the pore for pilin translocation are anchored in place, presumably by the peptidoglycan layer. These parts of the machinery may be either inactive (forming an empty pore without an associated pilus) or active, with associated motor proteins and a pilus that is in the process of either extension or retraction (Chang *et al.*^2016^). The activation of the type IV pilus machinery involves the recruitment to the pore of first the PilB and then the PilT motor proteins, needed to power the assembly, extension and retraction of a pilus (Fig. 3). In *M. xanthus*, motility reversals are accompanied by relocation of PilB and PilT from one pole to the other, a process that can be visualized by fluorescent protein tagging (Bulyha *et al.*^2009^). Rod-shaped bacteria therefore have a choice of two directions for motility, depending on which pole houses active type IV pilus machinery. By contrast, the spherical cells of *Synechocystis* are capable of movement in any direction (Schuergers *et al.*^2016^). Fluorescent protein tagging of PilB in *Synechocystis* shows that this protein tends to localize in one major patch at the cytoplasmic membrane, and the position of this patch strongly correlates with the direction of motility (Schuergers *et al.*^2015^). Motility switches in *Synechocystis* therefore likely involve the relocation of the motor ATPases, similar to rod-shaped bacteria except that the patch of active type IV pili can be located at any point on the cell surface.

Recently, type IV pili were shown to be crucial for the motility of the filamentous cyanobacterium *Nostoc punctiforme*, which produces differentiated motile filaments called hormogonia (Khayatan, Meeks and Risser 2015). In *N. punctiforme* hormogonia, the type IV pili are found at either end of each cell in the filament, close to the cell junctions. Motility reversals probably involve a switch in the active type IV pilus machinery from one end of each cell to the other, and this switch appears to be coordinated throughout the entire filament. However, no relocalization of PilB could be detected (Khayatan, Meeks and Risser 2015; Cho *et al.*^2017^).

A distinctive feature of type IV pilus-dependent motility is the requirement for a suitable surface for the pilus tips to adhere to. In many cases, the cells facilitate their own motility by laying down a track of secreted material, which is probably extracellular polysaccharide slime. This feature of the system promotes ‘social’ motility, since cells can follow the tracks that were laid down by their siblings (Li *et al.*^2003^; Burriesci and Bhaya 2008). Type IV pilus-dependent photomovement is therefore often observed as the concerted social movement (community phototaxis) of an entire colony towards or away from a light source (Burriesci and Bhaya 2008). Type IV pilus-dependent cyanobacterial photomovement is discussed further below. It appears to be under very complex control, and under different circumstances and in different model organisms it can show features of photokinesis, photophobic and scotophobic responses and true phototaxis. It can be involved in both single-cell and social behavior.

### The archaellum

Many archaea carry appendages superficially resembling the flagella of eubacteria. These appendages are protein fibers that drive swimming by rotation, and are described as flagella in older literature. However, despite the functional resemblance to flagella, the archaeal appendages are evolutionarily unrelated, and have therefore been renamed as ‘archaella’ (Albers and Jarrell 2015). In fact, the archaella are closely related to the type IV pili of eubacteria (described above), and also to the eubacterial type II secretion system, with many archaellum subunits showing obvious homology to components of both eubacterial systems. The distinction between an archaellum and a type IV pilus is that the archaellum drives motility by rotating, rather than by cycles of extension, surface binding and retraction as with the type IV pilus (Shahapure *et al.*^2014^; Albers and Jarrell 2015). Similarly to the type IV pilus, however, motility is powered by ATP hydrolysis by a motor protein located in the cytoplasm at the base of the structure. This contrasts with the flagellum, whose rotation is driven by transmembrane translocation of protons or Na^+^. The archaellum can power swimming in both directions, depending on the direction of rotation: cells swim forward when the archaella rotate clockwise, but backwards with counterclockwise rotation (Alam and Oesterhelt 1984). Therefore, photophobic or scotophobic responses in archaea result in 180° reversals in swimming direction, rather than a random choice of a new direction by tumbling as usually happens in flagellated bacteria. The mechanism of direction switching in archaella is poorly understood, although proteins that appear to be specifically involved in this process have been identified (Schlesner *et al.*^2009^). In other respects, the photophobic response of the archaeon *Halobacterium salinarum* is perhaps the most completely understood example of light-controlled motility in a prokaryote. In common with both proteobacteria and cyanobacteria, the *Halobacterium* system uses chemotaxis-like signaling proteins and a CheY-type response regulator to transmit a signal from the photoreceptor to the motility apparatus (Table 2), as discussed further elsewhere.

**Table 2.** Molecular systems for control of photobehavior in three prokaryotic species.

Organism	Motility system	Photosensor	Signal transduction system	Photobehavior
*Halobacterium salinarum* (Haloarchaeon)	Archaellum	Sensory rhodopsin I (orange/blue light)	MCP—CheA—CheW—CheY	Scotophobic/photophobic responses^a^
		Sensory rhodopsin II (blue light)	MCP—CheA—CheW—CheY	Photophobic response^a^
*Rhodobacter sphaeroides* (purple phototrophic bacterium)	Flagellum	Photosynthetic apparatus (blue, green and near IR light)	MCP—CheA—CheW—CheY	Scotophobic response^b^
*Synechocystis* sp. PCC6803 (cyanobacterium)	Type IV pilus	PixJ1 (cyanobacteriochrome; blue/green and possibly red light)	MCP—CheA—CheW—PATAN-CheY/CheY	Phototaxis (positive)^c^
		UirS (cyanobacteriochrome; UV/green light)	His-kinase/Response regulator/PATAN-CheY	Phototaxis (negative)^d^
		PixD (BLUF protein: blue light)	PATAN-CheY	Phototaxis (positive/negative)^e^
		Cph2 (cyanobacteriochrome; blue/green light)	c-di-GMP production	Motility inhibition^f^
		Cry-DASH (cryptochrome; blue light)	Unknown	Possible inhibition of negative phototaxis^g^
		Photosynthetic apparatus (blue, yellow and red light)	Unknown	Possible directional light sensor for phototaxis^h^

a Hoff, Jung and Spudich (1997)

b Armitage and Hellingwerf (2003)

c Bhaya, Takahashi and Grossman (2001)

d Song *et al.* (2011)

e Sugimoto *et al.* (2017)

f Savakis *et al.* (2012)

g Moon *et al.* (2010a)

h Schuergers, Mullineaux and Wilde (2017)

### Other motility mechanisms in phototrophic prokaryotes

Many diverse kinds of filamentous cyanobacteria are capable of ‘gliding’ motility on surfaces, and this gliding motility is linked to both phototaxis and chemotaxis. The mechanism(s) of gliding motility remain controversial, with ideas ranging from directional extrusion of polysaccharide slime to travelling surface waves and contractile fibrils at the cell surface (Häder 1987; Read, Connell and Adams 2007). There is no guarantee that all filamentous cyanobacteria move by the same mechanism, and some species may even use multiple mechanisms. Chemoheterotrophs use diverse gliding mechanisms, and some, such as *M. xanthus*, employ two distinct mechanisms of surface motility in the same cell, with one mechanism based on type IV pili (see above) and a second system known as ‘A-motility’ (Mauriello *et al.*^2010^). A-motility in *M. xanthus* appears to operate through mobile focal adhesion complexes (Mauriello *et al.*^2010^; Jakobczak *et al.*^2015^). By contrast, it was recently demonstrated that directional motility in the hormogonia of the filamentous cyanobacterium *N. punctiforme* employs a modified type IV pilus system (Khayatan, Meeks and Risser 2015) and therefore resembles the motility of unicellular cyanobacteria much more closely than had been thought (Wilde and Mullineaux 2015). This should prompt a re-examination of motility mechanisms in other filamentous gliding cyanobacteria, such as *Oscillatoria* species. Certain filamentous anoxygenic phototrophic prokaryotes, including the green non-sulfur bacterium *Chloroflexus aurantiacus*, are known to be capable of gliding motility (McBride 2001). The unicellular heliobacteria can also move by gliding, although some are flagellated (Beer-Romero, Favinger and Gest 1988). In these cases, the mechanisms of gliding motility remain obscure, as does their possible involvement in photobehavior. We are not aware of any demonstration of light-controlled motility in *Chloroflexus* or heliobacteria.

Many marine isolates of unicellular *Synechococcus* cyanobacteria are motile, and are capable of swimming at 5–25 μm s^−1^, despite lacking any conventional swimming appendages such as flagella. The mechanism remains unclear, but a plausible ‘surface wave’ model has recently been proposed, in which surface deformations are induced by a protein cargo travelling along a helical track at the cytoplasmic surface of the plasma membrane (Ehlers and Oster 2012). *Synechococcus* motility has been linked to chemotaxis towards sources of nitrogenous compounds, something that could clearly be advantageous in nutrient-poor regions of the ocean (Willey and Waterbury 1989). In contrast to chemotaxis, light-induced motility responses have never been observed in marine *Synechococcus* (Willey and Waterbury 1989) and it may be that the light environment in the oceans is too uniform on small length scales to make phototaxis worthwhile.

## SIGNAL TRANSDUCTION COMPONENTS INVOLVED IN PROKARYOTIC PHOTOBEHAVIOR

The signal transduction components that mediate between the photoreceptors and the motility apparatus are the most conserved and widespread part of prokaryotic photobehavior systems, in sharp contrast to the diversity of photoreceptors and motility machineries. For example, CheY-type response regulators are not only involved in chemotactic signal transduction systems but are also integral to many well-characterized examples of prokaryotic lifestyle decisions (He and Bauer 2014) as well as photobehavior control (Table 2). Second messengers such as cAMP and cyclic di-GMP (c-di-GMP) play similarly conserved roles in prokaryotic responses to environmental and intercellular signals (Pesavento and Hengge 2009). The conservation of the signal transduction components in such a wide range of prokaryotes hints at very deep evolutionary roots for prokaryotic behavior controlled by environmental sensing (Wuichet and Zhulin 2010). Sensors and motility systems have diversified or been independently recruited according to the many different environmental signals that are important for various prokaryotic lifestyles, and the distinct challenges of motility in diverse environments. However, there has been no need for such diversification in the parts of the system that do not directly interact with the outside world: the core signal transduction components in the prokaryotic cytoplasm. Prokaryotic signal transduction is generally mediated by simple one- and two-component systems and more complex sensory cascades such as those typically involved in chemotaxis which combine different input and output components (Ulrich, Koonin and Zhulin 2005; Wuichet and Zhulin 2010). Below we explain the principle of each kind of signal transduction system before discussing specific signal transduction components involved in light control of motility.

### One-component signal transduction

In one-component signal transduction systems, a single protein includes a sensory input domain fused to an output domain (Ulrich, Koonin and Zhulin 2005). A classic example is the LacI lactose operon repressor of *E. coli*, whose DNA association is modulated by the binding of inducer molecules (Lewis *et al.*^1996^). There are examples of one-component systems for control of photobehavior that fuse a photoreceptor protein to an output domain such as an enzyme responsible for production of a second messenger (Savakis *et al.*^2012^; Enomoto *et al.*^2014^) but we are not aware of any example of such a one-component system controlling a directional light response.

### Two-component signal transduction

In modular two-component systems, a sensor histidine kinase (which is often membrane bound) serves to detect an environmental signal. Activation of the histidine kinase results in autophosphorylation of a histidine residue, and subsequently the transfer of the phosphoryl group to an aspartate residue of the cognate response regulator (Stock, Robinson and Goudreau 2000). Phosphorylation of the response regulator usually activates the output domain, initiating the cellular response. If the response regulator is a DNA-binding protein, the output is typically a change in transcription. Response regulators which lack DNA-binding domains can control cellular responses by protein–protein or protein–RNA interactions or exert their function by enzyme activity (Galperin 2010). An example of a simple two-component system controlling photobehavior is known in cyanobacteria: the sensor is a membrane-integral photoreceptor that activates a DNA-binding response regulator when excited with UV light (Narikawa *et al.*^2011^; Song *et al.*^2011^; Ramakrishnan and Tabor 2016).

### Chemotaxis-like sensory cascades

Chemosensory signaling systems are a highly conserved feature of prokaryotic chemotaxis systems, and homologous systems are implicated in several examples of prokaryotic light-controlled movement (Yoshihara *et al.*^2000^; Bhaya, Takahashi and Grossman 2001; Hoff *et al.*^2009^). Chemosensory cascades employ methyl-accepting chemotaxis proteins (MCPs) to initiate downstream signal transduction in response to an environmental stimulus (Salah Ud-Din and Roujeinikova 2017). We refer to this class of proteins as MCPs throughout, as the term is specific and widely understood. The term is imperfect, however, not all MCPs are involved in motility control (Berleman and Bauer 2005) and some systems under discussion here are involved in phototaxis rather than chemotaxis (Yoshihara *et al.*^2000^; Bhaya, Takahashi and Grossman 2001; Hoff *et al.*^2009^). The role of MCPs is most comprehensively-understood in chemotaxis in *E. coli*, reviewed by Wadhams and Armitage (2004). In *E. coli*, homodimeric membrane-spanning MCPs bind molecules of a chemoattractant in the periplasm, either directly or by interacting with a periplasmic ligand-binding protein. A cytoplasmic adaptor protein called CheW links the cytoplasmic domain of the MCP to CheA, a dimeric histidine kinase. Decreases in ligand binding to the periplasmic domain of the MCP induce conformational changes that are transmitted across the membrane and promote trans-autophosphorylation of a histidine residue of CheA. CheA then transfers the phosphoryl group to an aspartate residue of CheY, a soluble response regulator that can diffuse through the cytoplasm to interact with the flagellar motor and provides the crucial link between the chemical sensor and the motility apparatus. CheA in *E. coli* can also phosphorylate CheB, a second soluble response regulator. CheB has a methylesterase domain that removes methyl groups from specific glutamate residues in the cytoplasmic domain of the MCP. The methylesterase activity is greatly stimulated by CheB phosphorylation, and therefore activation of the MCP by a decrease in ligand binding eventually results in decreased methylation of the MCP. This acts as a feedback mechanism for sensitization of the system to lower concentrations of attractant, decreasing the tendency of the MCP to activate CheA and thus eventually decreasing the phosphorylation of CheY (Wadhams and Armitage 2004). The methylation state of glutamate residues of the MCP plays a crucial role as the ‘memory’ of the system: it is this feature that enables the system to detect temporal changes in attractant concentration over a wide range of background concentrations. It should be noted that there is considerable diversity in chemosensory cascades of other bacteria, including alternative CheY phosphatases (Wuichet and Zhulin 2010).

Homologous motility control systems, including MCPs, CheA, CheW and CheY, are known to be involved in light control of motility in haloarchaea, cyanobacteria and purple photosynthetic bacteria, as discussed in detail below.

## PROKARYOTIC PHOTORECEPTORS

This section considers only direct photosensing systems: dedicated chromophore-binding proteins that undergo conformational change upon light absorption, thereby triggering signal transduction (Table 1). Prokaryotes also make use of various kinds of indirect photosensing (see Fig. 2 and discussion above) and some of these will be considered later in the discussion of photobehavior in specific groups of organisms.

### Sensory rhodopsins

Sensory rhodopsins are retinal-containing photoreceptors that are best characterized in haloarchaea (Luecke *et al.*^2001^). They are closely related to the energy-transducing bacteriorhodopsin and halorhodopsin, which act as light-powered transmembrane pumps for protons and chloride ions, respectively. Bacteriorhodopsin is best characterized in haloarchaea, but energy-transducing bacteriorhodopsin homologs (proteorhodopsins) are widespread in oceanic eubacteria (Béjà *et al.*^2001^). As compared to bacteriorhodopsin, a series of small sequence and structural differences in sensory rhodopsins are responsible for spectral tuning of the retinal chromophore and determining a function in signaling rather than energy transduction. Sensory rhodopsins are membrane-integral proteins with seven transmembrane alpha-helices that surround a covalently attached retinal molecule. Photoisomerisation of the retinal initiates a photocycle, driving conformational changes in the protein that initiate signal transduction (Luecke *et al.*^2001^). The haloarchaeon *H. salinarum* has two sensory rhodopsins (SR I and SRII) that are both employed to control photobehavior (Table 2). SR II acts as an intensity sensor for a photophobic response to blue light, whereas SR I induces a positive response to orange light as well as a photophobic response to blue light (Hoff, Jung and Spudich 1997). Each sensory rhodopsin has a cognate signal transducer (HtrI and HtrII) belonging to the family of MCPs. Sensory rhodopsins have also been detected and characterized in certain eubacteria, notably the halophilic chemoheterotroph *Salinibacter ruber* (Kitajima-Ihara *et al.*^2008^) and the filamentous cyanobacterium *Anabaena* sp. PCC 7120 (Vogeley *et al.*^2004^). However, sensory rhodopsins are only known to be involved in controlling motility in archaea (Hoff, Jung and Spudich 1997).

### Phytochromes and cyanobacteriochromes

Phytochromes and related photoreceptors were first characterized in plants, but later found to be widespread in cyanobacteria and other bacteria including the purple phototrophic bacterium *R. centenaria* (Kreutel, Kuhn and Kiefer 2010) and the chemoheterotrophs *Pseudomonas* species, *Deinococcus radiodurans* (Davis, Vener and Vierstra 1999), *Agrobacterium tumefaciens* (Lamparter *et al.*^2002^; Karniol and Vierstra 2003) and other species of the order *Rhizobiales* (Rottwinkel, Oberpichler and Lamparter 2010) (Table 1). The photosensory core module is fused to a variety of different output modules in these photoreceptors. All phytochrome-like photoreceptors have a bilin (linear tetrapyrrole) chromophore linked to one or two cysteine residues in a GAF (cGMP phosphodiesterase/adenylyl cyclase/FhlA) or PAS (period/ARNT/single-minded) domain. Based on domain architecture, Rockwell and Lagarias (2010) defined three bilin-binding photosensory modules: (i) red/far-red sensing classical phytochromes comprising a PAS-GAF-PHY (PHY: phytochrome-specific) module with a knotted architecture and a tongue region (Wagner *et al.*^2005^) (including plant and fungal phytochromes and biliverdin-binding bacteriophytochromes); (ii) unkotted GAF-PHY or GAF-GAF modules (Cph2 family) and (iii) cyanobacteriochromes characterized by a stand-alone photosensory GAF domain (Ikeuchi and Ishizuka 2008). Cyanobacteriochromes exhibiting extremely diverse photosensory characteristics are widespread in cyanobacteria and are often incorporated into large and complex multidomain proteins, sometimes with multiple chromophore-binding domains and signal-transducing modules (Yoshihara *et al.*^2000^; Rockwell and Lagarias 2010). Some cyanobacteriochromes are membrane-integral proteins, whereas others lack membrane-spanning domains and may be soluble or peripheral membrane proteins.

Absorption of a photon by the bilin chromophore of a phytochrome induces photoisomerization and a switch to a spectrally distinct form. In many photosensory proteins of the phytochrome lineage, both forms are stable and absorption of a second photon at a different wavelength is required to reverse the conformational change (Rockwell, Martin and Lagarias 2012). This feature of phytochromes makes them particularly suitable for sensing the relative intensity of light at the two wavelengths absorbed by the two photostates of the molecule. Those phytochromes that have rapid rates of dark reversion are more suited to the detection of absolute light intensity (Shinomura, Uchida and Furuya 2000; Rockwell, Martin and Lagarias 2016). Plant phytochromes sense exclusively red vs far-red light (Casal 2013). This is also true of many prokaryotic proteins belonging to the phytochrome family. However, cyanobacteriochromes may be tuned to regions of the spectrum anywhere from UV-A to far-red (Song *et al.*^2011^; Rockwell, Martin and Lagarias 2016). Cyanobacteriochromes are now known to be involved in many aspects of photobehavior. They can trigger signal transduction for photobehavior through all three of the routes discussed above: one-component systems, two-component systems and chemotaxis-like sensory cascades. Specific examples are discussed below.

### Sensors of blue light using FAD (BLUF) proteins

BLUF domain proteins (Table 1) were first discovered in purple bacteria, where a BLUF protein called AppA functions as a light-dependent anti-repressor protein controlling expression of photosynthesis genes (Gomelsky and Klug 2002). They are found in some eukaryotes as well as a wide variety of prokaryotes including *E. coli*, and are involved in regulation of gene expression, motility and biofilm formation (reviewed by Masuda 2013). A BLUF protein is known to regulate photophobic responses in the green alga *Euglena gracilis* (Iseki *et al.*^2002^), and the *Synechocystis* BLUF protein PixD is implicated in directional light sensing in this cyanobacterium (Masuda and Ono 2004), as discussed further below.

### Photoactive yellow protein (PYP)

PYP was first identified in the purple bacterium *Ectothiorhodospira halophila* as the photoreceptor regulating the blue light avoidance response (Sprenger *et al.*^1993^). The photochemistry of PYP is based on the chromophore *p*-coumaric acid, and its photocycle has been resolved in great detail (Changenet-Barret *et al.*^2007^). However, the downstream components of the signal transduction chain have not as yet been identified. In other purple bacteria which contain the *pyp* gene, its inactivation had no effect on photomovement, suggesting that PYP is not a general phototaxis photoreceptor (Kort *et al.*^2000^). PYP is sometimes found in fusion proteins such as the Ppr photoreceptor from *R. centenaria* which consists of a PYP domain, a bacteriophytochrome photosensory module and a histidine kinase output domain (Jiang *et al.*^1999^).

### Phototropins and LOV proteins

Phototropins are another photoreceptor type known from plants and algae. They consist of two chromophore-binding PAS domains followed by a serine/threonine kinase (Christie *et al.*^2002^). The PAS domains of phototropins belong to a subgroup of PAS domains which are found in proteins involved in light, oxygen and redox sensing, hence the name LOV (light, oxygen, voltage) for this domain (Lin 2002; Crosson, Rajagopal and Moffat 2003). Both LOV domains bind FMN as a cofactor, which upon blue light absorption binds a cysteine residue leading to a conformational change (Christie *et al.*^2002^). Blue light induces accumulation and light-avoidance responses of chloroplasts in plant cells. In plant species which have several chloroplasts per cell, plastids move towards weak light and move away from strong light illumination to avoid photodamage. This is controlled by phototropins (reviewed by Kong and Wada 2016). In green algae which have a single large chloroplast, the organelle rotates or moves within the cell to optimize light absorption, possibly also mediated by phototropins (reviewed by Suetsugu and Wada 2016). The two phototropins, phot1 and phot2, in *Arabidopsis* are localized at the plasma membrane. Phot2 mediating the avoidance response is also localized at the outer chloroplast envelope, resembling the model for cyanobacterial phototaxis (see below). No phototropins have been identified in cyanobacteria. However, cyanobacterial proteins containing LOV domains have been identified (Crosson, Rajagopal and Moffat 2003; Cao *et al.*^2010^). One such protein controls levels of the second messenger c-di-GMP in response to blue light, potentially influencing biofilm formation (Cao *et al.*^2010^).

### Cryptochromes

Cryptochromes are blue light flavoprotein photoreceptors that are very widespread, being found in bacteria, plants and also in non-phototrophic eukaryotes (Table 1) (Ahmad and Cashmore 1993). In plants, they regulate developmental processes and the circadian clock (Liu *et al.*^2011^). In animals from flies to mammals, cryptochromes are important components of the circadian clock (Chaves *et al.*^2011^). They show homology to photolyases, enzymes which repair DNA in a light-dependent manner. It was initially proposed that eukaryotic cryptochromes derived from an ancestral bacterial photolyase. However, characterization of a photolyase-like protein from the cyanobacterium *Synechocystis* (Cry) revealed a new type of cryptochromes in prokaryotes, named the Cry-DASH (*Drosophila, Arabidopsis, Synechocystis, Human*) family because homologs of this group can be identified in these organisms (Brudler *et al.*^2003^). In contrast to cryptochrome photoreceptors of plants and animals, the members of the Cry-DASH family were shown to have a residual repair activity for single-stranded DNA. Members of all cryptochrome classes bind FAD non-covalently as the chromophore for absorption of blue light. In addition, photolyases and all cryptochromes use a second cofactor (e.g. methenyltetrahydrofolate or pterin) as an antenna chromophore (Kiontke *et al.*^2014^). The *Synechocystis* Cry-DASH protein has been suggested to be involved in the UV-A/blue light phototaxis response, based mainly on indirect measurements (Moon *et al.*2010b).

## LIGHT-CONTROLLED MOTILITY IN HALOARCHAEA

Currently, the best understood prokaryotic photosensory system involved in light-dependent motility is probably the sensory rhodopsin system of *H. salinarum* (Table 2). This system involves the photoreceptors sensory rhodopsins SR I and SRII and the signal transducers HtrI and HtrII, which are classical MCPs. Both sensory rhodopsins bind a retinal chromophore and transmit the light signal to the cognate MCP. The Htr proteins form a complex with typical CheA and CheW chemotaxis proteins, which control the activity of CheY via a phosphorylation cascade (Hoff, Jung and Spudich 1997; Armitage and Hellingwerf 2003). Phosphorylated CheY then regulates the direction of archaella rotation, although the mechanism by which phospho-CheY interacts with the archaellar motor is not known. Some possible adaptor proteins have been identified (Schlesner *et al.*^2009^). SRII absorbs blue light, which generates a photocycle with several intermediates. In their active state, they induce the autophosphorylation of CheA resulting in CheY phosphorylation and a motility reversal resulting in movement away from increasing intensities of blue light. SR I in its ground state absorbs an orange photon and induces positive movement towards a light source, whereas a long-lived photointermediate of SR I absorbs a blue photon and sends a negative signal. Cells accumulate away from a blue light source, a typical photophobic response. *Halobacterium* has a CheC homolog that may terminate the response by removing the phosphoryl group from CheY, and it has a typical CheR methyltransferase and CheB methylesterase (Perazzona and Spudich 1999). Analogy with the *E. coli* chemotaxis system would suggest that CheB and CheR could play a role in the ‘memory’ of the system by tuning the methylation states of HtrI and HtrII according to prevailing light levels, thus adjusting the sensitivity and increasing the dynamic range of the system. Photostimulation of *Halobacterium* does indeed induce changes in the methylation states of HtrI and HtrII, and mutant phenotypes are consistent with the idea that methylation regulates the responsiveness of the system to stimulation. However, there are some differences from the *E. coli* system, notably that transducer methylation is a global response that is not specific to the transducer through which the stimulus was sent (Perazzona and Spudich 1999). See Schlesner *et al.* (2012) for a recent update on the *Halobacterium* taxis signal transduction system. It should be noted that there is evidence for an additional system for control of photomovement in *Halobacterium*, triggered by changes in membrane potential generated by the energy-transducing bacteriorhodopsin (Grishanin *et al.*^1996^). There is also evidence that activation of the sensory rhodopsins stimulates production of fumarate, and fumarate concentration acts as an additional control on flagellar motor switching (Marwan, Schäfer and Oesterhelt 1990).

## LIGHT-CONTROLLED MOTILITY IN ANOXYGENIC PHOTOTROPHIC BACTERIA

The anoxygenic phototrophic bacteria are extremely diverse. Many species are motile, but light-controlled movement has been characterized only in flagellated purple photosynthetic bacteria. Two distinct kinds of photobehavior have been characterized, and these are discussed in the sections below.

### Indirect photosensing for scotophobic responses in purple bacteria

Motile purple bacteria such as *R. sphaeroides* (Table 2) exhibit scotophobic behavior in which they respond to decreasing intensities of favorable illumination by triggering motility reversals. Although such bacteria contain several different photoreceptors, it appears that photomovement is controlled by indirect light sensing by the photosynthetic apparatus. Classic early experiments by T.W. Engelmann, described by Drews (2005) showed that a phototrophic bacterium (most probably a *Chromatium* species) accumulated in regions illuminated by infrared light between 800 and 900 nm, corresponding to an absorption peak of bacteriochlorophyll. Later experiments showed that photosynthetic electron transfer was essential for photoresponses of *R. sphaeroides* and other purple bacteria. Mutants lacking the photosynthetic reaction center were able to move in response to chemical but not to light signals (Armitage and Evans 1981). Inhibition of the photosynthetic electron transport chain also led to an inhibition of photoresponses, and further experiments applying different light intensities suggest that *R. sphaeroides* cells respond to a change in light intensity only in the range where photosynthesis is not saturated (Grishanin, Gauden and Armitage 1997). Photosynthetic electron transport results in changes in the redox state of electron carriers, the generation of a transmembrane proton gradient and more downstream effects such as changes in ATP levels and metabolite pools. Any of these could potentially be the source of the signal controlling photobehavior, but so far, the link is not clear. Two typical chemotaxis signal transducers, CheA and CheW, were shown to be essential for photoresponses (Romagnoli and Armitage 1999). This makes it very likely that an MCP is involved in collecting the signal from the photosynthetic apparatus, and that a CheY transmits the signal to the flagellar motor (Table 2). So far, it is not clear which of the 13 *R. sphaeroides* MCPs and 6 CheY proteins might be specifically involved in photobehavior (Armitage and Hellingwerf 2003). The scotophobic response of *R. sphaeroides* requires a ‘memory’ for the prevailing light conditions which likely involves MCP methylation. The *R. sphaeroides* genome sequence reveals three CheR methyltransferases and two CheB methylesterases, but their precise roles remain to be determined (Martin *et al.*^2001^; Armitage and Hellingwerf 2003). Photobehavior in *R. sphaeroides* very likely shares components and pathways with chemotaxis, and this may facilitate the integration of light and chemical signals. For example, it is known that there is cross-talk between photoresponses and aerotaxis influenced by oxygen levels: photophobic responses are somewhat inhibited in the presence of oxygen. This could be explained by a single signal transduction system responding to the redox state of a component of the interacting photosynthetic and respiratory electron transport chains (Armitage 1997).

### True phototaxis in *Rhodospirillum centenum*

The purple bacterium *R. centenaria* shows true phototactic behavior in the form of community phototaxis when swarming in colonies on agar surfaces (Ragatz *et al.*^1994^, ^1995^). Cells under these conditions have large numbers of peritrichous flagella. The cells move towards light between 800 and 850 nm, a wavelength region overlapping with the absorption peaks of the bacteriochlorophyll-containing light harvesting and reaction center complexes. For this response, light detection likely depends on photosynthetic activity, as for *R. sphaeroides*. Negative phototaxis is induced by light at wavelengths below 600 nm (Ragatz *et al.*^1995^). The cell colonies move directly towards or away from a single directional light source, and can also integrate the information from two light sources, for example, moving towards a point midway between two light sources at 45° to each other (Ragatz *et al.*^1995^). Two potential signal receptors have been identified for *R. centenaria* (Table 1). The first is an MCP called Ptr, which most probably detects a signal generated by the photosynthetic apparatus and feeds it into the chemosensory pathway. Ptr was shown to be essential for negative and positive phototactic responses of *R. centenaria* but not for chemotaxis (Jiang and Bauer 2001). It is proposed that Ptr, which contains a putative heme-binding site, senses the redox state of cytochrome *c* of the electron transport chain and transduces this signal to components of the chemotaxis system. This would be an example of indirect photosensing (Fig. 2). The second signal receptor is a photoreceptor called Ppr which contains PYP and bacteriophytochrome photoreceptor domains (see above) linked to a histidine kinase domain and is involved in regulation of pigment synthesis (Jiang *et al.*^1999^). There is evidence for the interaction of Ppr with chemotaxis proteins, although its mutation has no obvious effect on phototactic behavior (Kreutel, Kuhn and Kiefer 2010). *In vivo* and *in vitro* experiments showed that the sensor protein interacts with CheW and may form a complex with the CheAY protein (Kreutel, Kuhn and Kiefer 2010). However, it remains unexplained how cells regulate the switch between negative and positive phototactic colony movement. More fundamentally, the mechanism of light-direction sensing is unknown. *R. centenaria* shows a scotophobic response but seems to be unable to sense light direction when cells are dispersed in liquid media, which suggests that direction sensing depends on interactions between cells packed into a dense colony (Sackett *et al.*^1997^). It remains unclear whether these interactions consist of a simple combination of shading and blocking, or whether the mechanism of community phototaxis is subtler. A simple model based on the scotophobic response, whereby cells just seek to avoid being shaded by their neighbors, would not explain why the colony moves in a concerted fashion towards the light source without spreading laterally.

## LIGHT-CONTROLLED MOTILITY IN CHEMOHETEROTROPHIC BACTERIA

Blue light can influence the motility of *E. coli* cells (Yang, Inokuchi and Adler 1995). Upon exposure to blue light, some enterobacteria show photophobic responses, tumbling or even swimming away from the light source and then completely losing motility after longer illumination. An *E. coli* mutant in the gene encoding the ferrochelatase enzyme showed a tumbling response at much lower intensities of blue light (10 μmol photons m^−2^ s^−1^, 396–450 nm) than the wild type does (Yang, Inokuchi and Adler 1995). The ferrochelatase catalyzes the synthesis of heme from the substrate protoporphyrin IX, which accumulates in cells deficient in ferrochelatase activity. The authors suggest that the accumulation of this photosensitizer leads to the production of reactive oxygen species, which might be the signal for tumbling. They also show that components of the chemotaxis signal transduction chain are involved in this process. In wild-type cells, the blue light-induced tumbling response is achieved only by the application of strong blue light, which then after longer exposure results in a permanent loss of motility. Wright *et al.* (2006) used a more balanced illumination in order to discriminate between blue light as a repellent signal and motor damage by high irradiation. The action spectrum of the repellent response suggested a flavin as the chromophore. Furthermore, they demonstrated that the FAD-binding aerotaxis receptor Aer and the chemotaxis receptor Tar were involved in blue light responses of *E. coli* cells, but they also suggest an additional, so far unknown, photosensor, which absorbs the blue light stimulus and controls the adaptation process. More recently, it was shown that *E. coli* encodes a blue light-absorbing BLUF-domain protein YcgF (Tschowri, Busse and Hengge 2009). This protein was shown to be involved in regulation of biofilm formation but is not known to be involved in the above-mentioned blue light effect on motility.

A second characterized example of photobehavior in a chemoheterotrophic bacterium is a UV-induced response in *Bacillus subtilis* in which exposure to UV-A and UV-B light induces cells to migrate to the edge of colonies, forming a ring pattern (Delprato *et al.*^2001^). The authors suggest that the UV exposure triggers chemotactic movement away from the waste products of the cells. This could account for the ring-shaped colonies formed under these conditions, although the physiological advantage is not clear. *Bacillus subtilis* has a phototropin-like photoreceptor, YtvA (Avila-Pérez, Hellingwerf and Kort 2006; Gaidenko *et al.*^2006^), that could potentially be involved in triggering the response.

## LIGHT-CONTROLLED MOTILITY IN CYANOBACTERIA

Cyanobacteria show an exceptionally complex range of photoresponses, with some species such as the unicellular model cyanobacterium *Synechocystis* showing photokinesis as well as true positive and negative phototaxis (Table 2) and exhibiting sophisticated responses to multiple light signals (Chau, Bhaya and Huang 2017; Schuergers, Mullineaux and Wilde 2017). The reasons why cyanobacteria have evolved a suite of photoresponses that appears so complex in comparison to other phototrophic prokaryotes are not certain. However, the necessity for such complex behavior could be related both to the greater complexity of the cyanobacterial photosynthetic apparatus with its two photosystems working in tandem (Blankenship and Hartman 1998) and to the danger of photodamage when performing photosynthesis in the presence of oxygen (Szabó, Bergantino and Giacometti 2005). Cyanobacteriochromes are involved in many of the better-understood cyanobacterial photoresponses, although cyanobacteria also contain several other classes of photoreceptors and the photosynthetic electron transport chain is potentially an additional source of signals for photobehavior.

### Light regulation of motility vs sessility in unicellular cyanobacteria

Two well-characterized blue light-dependent photoresponses in unicellular cyanobacteria control switches between different lifestyles. In both cases, the photoreceptor is a cyanobacteriochrome that triggers production of the second messenger c-di-GMP when activated by blue light.

In *Synechocystis*, the four-color cyanobacteriochrome (CBCR) sensor Cph2 has two photosensory domains that are fused to enzymatic domains related to synthesis (GGDEF) and degradation (EAL) of the second messenger c-di-GMP. The photoreceptor consists of six domains in the sequence GAF-GAF-GGDEF-EAL-CBCR-GGDEF. The N-terminal module comprises an unknotted GAF-PHY phytochrome photoreceptor module, which absorbs in the red/far-red light region with the absorption maximum being blueshifted compared to canonical phytochromes (Park *et al.*^2000^; Wu and Lagarias 2000; Anders *et al.*^2011^), whereas the C-terminal CBCR module absorbs in the green/blue spectral region (Savakis *et al.*^2012^). The C-terminal domain covalently binds the bilin chromophores phycocyanobilin (PCB) or phycoviolobilin (PVB) via two cysteine residues and activates the adjacent GGDEF domain under blue light. The module then produces c-di-GMP, which inhibits type IV pilus-dependent motility in *Synechocystis*. The mechanism is not yet known, but it was predicted that *Synechocystis* PilB1 might bind c-di-GMP (Wang *et al.*^2016^) suggesting a direct effect of c-di-GMP on the motility apparatus. The c-di-GMP content of *Synechocystis* is indeed higher under blue light, when compared to green, red and white light illumination (Agostoni *et al.*^2013^; Angerer *et al.*^2017^). Interestingly, the difference in the c-di-GMP concentration between green and blue light-grown cells is just 50%, which is in good agreement with *in vitro* measurements of the light-dependent enzymatic activity of CBCR-GGDEF module of Cph2 (Savakis *et al.*^2012^). It appears that the differences in overall cellular c-di-GMP content between motile and non-motile states are quite small. Therefore, localized pools of this second messenger may be significant, as in other bacteria (Jenal, Reinders and Lori 2017).

The GGDEF domain in the N-terminal part of Cph2 is most probably not functional because it contains a degenerated GGDEF motif, so it is possible that c-di-GMP binds to this domain. Savakis *et al.* (2012) showed that the EAL domain of Cph2 is active, as the GAF-GAF-GGDEF-EAL part can complement the effect of cellular high c-di-GMP content. Expression of this module allows the cells to move towards blue light and it can also compensate for the high c-di-GMP content, which is produced upon expression of the blue/green CBCR-GGDEF module. However, the biological function of the N-terminal red/far-red absorbing photosensory module is still poorly understood. The functional competence of the red/far-red light switching Cph2 photosensory domain was corroborated by previous work (Fiedler *et al.*^2004^) where it was shown that *Δcph2* mutant cells grew slower under red light, indicating that the far-red absorbing state may be the signaling state of the photoreceptor. Indeed, the far-red absorbing state of the recombinant GAF-GAF fragment is unstable, decaying in darkness with a half-life of 54 min (Anders *et al.*^2011^). An interaction of the EAL and GGDEF domains of Cph2 with another c-di-GMP synthesizing enzyme, Slr1143, was recently demonstrated, although this interaction is not light-dependent *in vivo* (Angerer *et al.*^2017^). Nevertheless, *Δslr1143* mutant cells show altered motility behavior, moving towards a high-intensity red light source under conditions where the wild type is non-motile (Angerer *et al.*^2017^).

In the closely related thermophilic unicellular cyanobacteria *Thermosynechococcus elongatus* and *Thermosynechococcus vulcanus*, SesA is a blue-green photoreversible cyanobacteriochrome with blue light-induced diguanylate cyclase activity (Enomoto *et al.*^2014^). The domain structure of SesA is simpler than that of *Synechocystis* Cph2, as it possesses only one CBCR-GAF domain fused to a c-di-GMP-producing GGDEF domain. SesA was shown to be essential for blue light-induced aggregation of *T. vulcanus*. This aggregation depends on production of extracellular cellulose. Enomoto *et al.* (2014) were able to propose a complete signal transduction chain for the response, starting with blue light activation of SesA and production of c-di-GMP by the activated GGDEF domain of SesA, followed by c-di-GMP binding to a PilZ domain to activate Tll007, a membrane-integral cellulose synthase that exports cellulose into the cell surface layer, where it promotes cell aggregation.

The physiological advantages of a switch to a sessile lifestyle in blue light are not clear. In the case of *Thermosynechococcus* cell aggregation, there may be some benefit derived from self-shading to minimize blue light-induced damage to the oxygen-evolving Mn cluster of photosystem II (Enomoto *et al.*^2014^). In the case of *Synechocystis*, blue light-induced motility inhibition defies simple explanation, since it might be imagined that it would trap cells in a damaging light environment. However, *Synechocystis* has a suite of other light-induced motility responses, some of which are discussed below. The role of Cph2 probably has to be understood as one of many light and chemical inputs into a complex decision-making process. It appears that enhanced c-di-GMP production is not a universal cyanobacterial response to blue light, since the non-motile cyanobacterium *Synechococcus elongatus* has a protein in which a blue light receptive LOV domain is fused to EAL and GGDEF motifs. This protein shows enhanced c-di-GMP phosphodiesterase activity upon blue light activation, which would imply that the c-di-GMP level decreases in response to blue light in this cyanobacterium (Cao *et al.*^2010^).

### Phototaxis in unicellular cyanobacteria—principles

**Figure 4.**
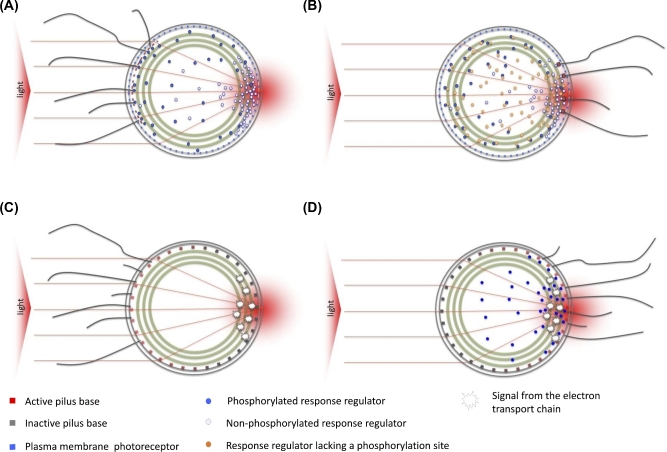
Hypothetical models for directional light perception in *Synechocystis*, leading to positive (**A,C**) or negative (**B,D**) phototaxis. All models depend on light focusing by the cell for directional light perception. (**A, B**) Directional light perception by plasma membrane photoreceptors (Schuergers *et al.*^2016^). Positive phototaxis (**A**) depends on a directional light perception by a photoreceptor such as PixJ1 in the plasma membrane, and activation of pilus bases by binding a CheY-type response regulator, by analogy with models for *Pseudomonas* (Bertrand, West and Engel 2010). PixJ1 in the focused light spot is photoactivated, resulting in local phosphorylation of its cognate response regulator. Phosphorylation weakens binding of the response regulator to the pilus base, resulting in local inactivation of pilus extension. PilB1 relocates to the opposite side of the cell, resulting in movement towards the light source (Schuergers *et al.*^2015^). Negative phototaxis (**B**.) is triggered by the presence of a non-phosphorylatable response regulator such as LsiR or PixE (Song *et al.*^2011^; Sugimoto *et al.*^2017^), which prevents the binding of the non-phosphorylated response regulator to the pilus base. Pilus activity can then be triggered only by low-affinity binding of the phosphorylated response regulator, which is locally generated by activated photoreceptors. (**C, D**) An alternative scenario in which the directional signal comes from local excitation of the photosynthetic apparatus in the thylakoid membranes. The specific photoreceptor systems (PixJ1, UirS, PixD) do not provide directional signals but instead tune the system for positive or negative phototaxis by controlling the availability of response regulators (Schuergers, Mullineaux and Wilde 2017).

The unicellular cyanobacteria *Synechocystis* and *T. elongatus* are both capable of true phototaxis when moving by type IV pilus-dependent motility on surfaces. Only positive phototaxis has been observed in *T. elongatus* (Kondou *et al.*^2001^), but *Synechocystis* exhibits both positive and negative phototaxis depending on the wavelength and intensity of light (Bhaya 2004). Phototaxis in both organisms is normally observed as the social movement of colonies of cells on an agar or agarose surface illuminated from one side, similar to *R. centenaria* (see above). Also similar to *R. centenaria*, phototaxis in *Synechocystis* and *T. elongatus* is not a response to a light gradient or a temporal change in light intensity, rather it involves direct sensing of the position of a light source. However, in contrast to *R. centenaria* (Sackett *et al.*^1997^) light-direction sensing in *Synechocystis* has been shown *not* to require the dense packing of cells into a colony. When motile *Synechocystis* cells are spread out on a suitable surface so that they are no longer in contact with other, individual cells are still capable of phototaxis (Choi *et al.*^1999^; Burriesci and Bhaya 2008; Chau *et al.*^2015^; Schuergers *et al.*^2016^). Individual cells can control their motility so as to move towards a light source with an accuracy of about ±15°, and the orientation of motility takes about 1 min to complete (Schuergers *et al.*^2016^). As explained above, the basis for directional light perception at the single-cell level is the ability of the spherical *Synechocystis* cell to act as a microlens. Light shone from one side is focused to a sharp spot at the opposite side of the cell (Schuergers *et al.*^2016^). For positive phototaxis, this gives rise to the model illustrated in Fig. 4 in which a photoreceptor evenly distributed around the cell periphery (most probably in the cytoplasmic membrane) responds to the bright spot of focused light, triggering a highly localized signal transduction pathway which locally inactivates the type IV pilus apparatus. By analogy with the mechanism of motility reversals in *M. xanthus* (Bulyha *et al.*^2009^), inactivation probably occurs by decoupling of the motor proteins PilB and PilT. Type IV pilus activity is therefore confined to the opposite side of the cell (the side facing the light source), and the cell moves towards the light. Negative phototaxis could be explained by a similar model, except that in this case the photoreceptor would trigger localized activation of the type IV pili (Nakane and Nishizaka 2017). The key conditions for this model to work are as follows:

- The photoreceptors should be located close to the periphery of the cell.
- The photoreceptors should be rather evenly dispersed around the cell periphery. Otherwise cells would not have their observed ability to detect and respond to illumination from any direction.
- Signal transduction between the photoreceptor and the motility apparatus should be localized. At a maximum, the messenger molecule should not diffuse more than 90° around the cell periphery before interacting with a pilus motor.
- However, rapid signal transduction is not required, as the timescale for direction switching is slow (about 1 min).

Single-cell phototaxis is less well characterized in *T. elongatus*. This species has elongated rod-shaped cells. There is evidence that a *T. elongatus* phototaxis photoreceptor is clustered at the cell poles (Kondou *et al.*^2002^) as discussed further below. By analogy with rod-shaped chemoheterotrophs such as *M. xanthus* that exhibit type IV pilus-dependent motility (Bulyha *et al.*^2009^), directional control is likely to involve selective activation/inactivation of the motility apparatus at the two cell poles. For a model similar to the *Synechocystis* one to be applied to *T. elongatus*, the cell would need a method to concentrate light at the pole furthest away from the light source. The optical properties of *T. elongatus* need further investigation.

### Phototaxis in unicellular cyanobacteria—signal transduction components

Much work on phototaxis in unicellular cyanobacteria has gone into identifying and characterizing the photoreceptors and other signal transduction components involved (Table 2; Fig. 3). The best-characterized organism is *Synechocystis*, and the picture that emerges is complex. The phenotypes that result from deletion of specific photoreceptors or signal transduction components generally do not consist of random motility in directional illumination, rather the direction of motility tends to be reversed as compared to the wild type (Bhaya 2004). This suggests that there are at least two independent systems for photosensing and signal transduction, and that these systems compete to determine whether phototaxis is positive or negative. Which system ‘wins’ depends on their relative excitation with the intensity and spectral quality of light used in a specific experiment, probably combined with other factors such as the prior acclimation of the cells. Unfortunately, the mutagenesis experiments that demonstrate the involvement of specific photosensory systems in control of phototaxis do not clearly establish the precise roles of the different systems. For example, if loss of a particular photosensor represses positive phototaxis, it could be for two different reasons:

- The photosensor is required for directional light sensing for positive phototaxis (as discussed by Bhaya, Takahashi and Grossman 2001).
- The photosensor is responsible for longer-term light adaptation of the system, operating in light-dependent control of the expression or activity of other components that promote positive or negative phototaxis (as discussed by Song *et al.*^2011^ and Sugimoto *et al.*^2017^).

Although the involvement of several specific photoreceptors in either (I) or (II) has been established, it is not yet certain which (if any) of the photoreceptors are responsible for directional light perception. It remains possible that all the characterized photoreceptors are responsible only for longer-term tuning of the system to favor positive or negative phototaxis (Sugimoto *et al.*^2017^). In this case, the actual directional light signal would have to come from somewhere else, probably from the activity of the photosynthetic apparatus generating localized signals that are transmitted to the motility apparatus (Fig. 4). It has been argued that phototaxis probably does not depend on signals generated by the photosynthetic apparatus, because phototaxis is not abolished by the photosystem II inhibitor DCMU (Choi *et al.*^1999^). However, this experiment does not exclude some possibilities, including redox signals at the acceptor side of photosystem I, which can still be generated when photosystem II is blocked (Mullineaux 2014). Further work is needed for definitive identification of the directional sensors, including studies of the localization and interactions of the photoreceptors and their associated signal transducers, and further studies on the possible role of the photosynthetic apparatus in generating directional signals.

The first *Synechocystis* phototaxis locus to be identified was the *tax*1 gene cluster. Disruption of *tax1* results in negative phototaxis under conditions when the wild type shows positive phototaxis (Yoshihara *et al.*^2000^; Bhaya, Takahashi and Grossman 2001; Ng, Grossman and Bhaya 2003). The gene cluster includes the *pixJ1* (*taxD1*) gene encoding a cyanobacteriochrome photoreceptor. PixJ1 has two transmembrane domains, two GAF domains and a C-terminal MCP module. Proteomic studies show that PixJ1 is located in the *Synechocystis* plasma membrane (Pisareva *et al.*^2007^), thus fulfilling one of the required conditions for a directional light sensor (see above). The chromophore PCB is only bound to the second GAF cyanobacteriochrome domain, and the protein isolated from *Synechocystis* shows reversible photoconversion between blue (P_b_) and green (P_g_) absorbing states, where the P_b_ form was stable in the dark (Yoshihara *et al.*^2004^, ^2006^). Therefore, blue light illumination leads to the conversion into the unstable form P_g_. As the *pixJ1* mutant shows negative phototaxis also in response to yellow and red light, the authors speculate that the dark-stable P_b_ form is active and induces positive phototaxis, whereas the Pg form which is formed after blue light illumination is not able to induce positive phototaxis, which would explain why the cells do not show any motility response under blue light. However, Δc*ph2* mutant cells, which move under blue light due to a low c-di-GMP content, show positive phototaxis towards blue light (Wilde, Fiedler and Börner 2002). Thus, PixJ1 can induce also positive phototaxis under blue light. It is not clear so far how this blue/green photoreceptor can change the direction of movement under so many different light colors. However, reconstitution of PixJ1 with different chromophores in *E. coli* indicates that it has the potential to bind biliverdin in place of the usual PCB chromophore (Yoshihara *et al.*^2006^), which could potentially allow a proportion of PixJ1 proteins to act as red light sensors. It should be noted that the identity of the chromophore bound *in vivo* is often unclear for bilin-binding proteins (Yoshihara *et al.*^2006^). Interestingly, a PixJ1 homolog, TePixJ from *T. elongatus*, shows polar localization in this motile rod-shaped cyanobacterium (Kondou *et al.*^2002^) supporting the idea of PixJ1 as a sensor of light direction.

The *Synechocystis tax1* gene cluster encodes a classic set of MCP-associated signal transduction components (Bhaya, Takahashi and Grossman 2001). In addition to the MCP module that forms an integral part of PixJ1, there are CheW and CheA homologs (PixI and PixL, respectively), and two response regulators (PixG and PixH) that can be classified as CheY-like because their genes form part of a taxis operon. Like several other cyanobacterial response regulators implicated in motility control, PixG belongs to a subfamily carrying a PATAN (PatA N-terminal) domain in addition to the CheY receiver domain (Makarova *et al.*^2006^) (Fig. 3). The function of the PATAN domain is unknown, but a direct or indirect role in interaction with the cyanobacterial motility apparatus is plausible because a single PATAN-domain response regulator is found in all three *tax* operons. By analogy with *E. coli* chemotaxis and *Halobacterium* photomovement (both discussed above), it is likely that light activation of PixJ1 induces a change in the phosphorylation state of PixL, although it is not certain which photostate of the cyanobacteriochrome promotes activation of the MCP and PixL autophosphorylation. Phosphorylated PixL would then transfer the phosphoryl group to the CheY-type response regulators PixH and PixG. One or both of these response regulators might then be involved in transmitting the signal to the type IV pilus motors. The model discussed in the previous section (Fig. 4A) predicts that positive phototaxis will result from localized deactivation of type IV pilus motors in the vicinity of a PixJ1 photoreceptor that has been stimulated by the bright focused spot of light. However, it is not known whether the response regulators directly interact with the type IV pilus motor proteins. Further studies of the localization and interactions of PixH and PixG are required. It is interesting that *Synechocystis* has no homologs of any known signal-terminating CheY phosphatase, suggesting that the signal is terminated by spontaneous dephosphorylation of the CheY-type response regulator. Typically, spontaneous CheY dephosphorylation has a half-time of seconds (Wadhams and Armitage 2004), and this would be sufficient in *Synechocystis*, where motility reversals occur on slow timescales of about 1 min. The *Synechocystis* genome contains two additional gene clusters (*tax2* and *tax3*) that show strong homology in content and organization to *tax1*. However, the MCP-like proteins of *tax2* and *tax3* differ from PixJ1 in that they lack the cyanobacteriochrome domain, and therefore are probably not photosensors. The proteins encoded by *tax2* and *tax3* may be responsible for motility control in response to unknown chemical or mechanical signals, in addition to the light control provided by *tax1. P. aeruginosa* provides a precedent for mechanosensing of surface attachment (Persat *et al.*^2015^; Rodesney *et al.*^2017^).

Another cyanobacteriochrome implicated in directional light sensing in *Synechocystis* is the UV-A photosensor UirS (Slr1212), which also exhibits an ethylene-binding domain (Song *et al.*^2011^; Lacey and Binder 2016). Unlike PixJ1, this multidomain protein has no MCP-like domain, but it is a classic two-component system sensor kinase. Knockout studies indicate that UirS and its downstream response regulators UirR and LsiR are required for negative phototaxis in response to unidirectional UV-A light (Song *et al.*^2011^). UirS has three predicted membrane-spanning domains, and, like PixJ1, it is an integral component of the plasma membrane (Kwon *et al.*^2010^). The UirS-cyanobacteriochrome domain possesses a dual-linked PVB chromophore and photoconverts between UV-absorbing (P_uv_) and green-absorbing (P_g_) states. According to the model proposed by Song *et al.* (2011), the response regulator UirR is phosphorylated by UirS upon UV irradiation. The active UirR transcription factor then binds to the promoter of the sRNA CsiR1, also driving the expression of the LsiR PATAN-CheY response regulator gene (Ramakrishnan and Tabor 2016). LsiR is implicated in negative phototaxis since its constitutive expression leads to negative phototaxis even under red light and could complement the UV phototaxis phenotype in mutants lacking UirS and UirR (Song *et al.*^2011^). These data suggest that the function of UirS in the negative phototaxis response is to activate LsiR expression: UirS itself may not be directly involved in directional light sensing (Song *et al.*^2011^). Slightly different data were published by Narikawa *et al.* (2011) on UirS, which they named PixA. This group used two wild-type *Synechocystis* strains with different genetic backgrounds (Kanesaki *et al.*^2012^). Cells of the PCC-P strain show positive phototaxis, whereas the PCC-N strain was always negatively phototactic. In the PCC-P strain, the UirS mutation led to inversion of the response: the cells moved away from white light. No effect was seen in UirR and LsiR mutants. In contrast, in the PCC-N background UirS had no effect, whereas inactivation of UirR and LsiR led to a switch from negative to positive phototaxis. The phototactic behavior of *Synechocystis* seems to be regulated by factors whose balance determines the direction of movement towards or away from a light source (Narikawa *et al.*^2011^).

UirS is also an ethylene receptor, and therefore in some publications it is named SynETR1 based on the well-described ethylene receptors in plants (Lacey and Binder 2016). These authors showed that UirS can directly bind ethylene, thereby leading to an influence of ethylene on motility and photobehavior. Application of ethylene accelerated the movement of *Synechocystis* cells on agar plates. The cells moved faster, and also the proportion of motile cells increased upon ethylene exposure (Lacey and Binder 2016). Deletion of the second ethylene-binding transmembrane domain of UirS led to a greater enhancement of the motility response (Lacey and Binder 2016). Taken together, inactivation of UirS, UirR, LsiR and ethylene application had the same effects on phototaxis: both strategies led to an enhancement of positive phototaxis. This could suggest that ethylene inhibits the phosphorylation of UirR in response to UV activation of UirS, thereby downregulating LsiR expression and inhibiting negative phototaxis. This in turn would accelerate movement towards light. The physiological benefit of ethylene control of photobehavior is unclear. Ethylene may originate from decomposition of organics in sunlight and thus could be an indicator of light for photosynthesis. Ethylene-producing enzymes are also present in many prokaryotes and eukaryotes which might be associated with cyanobacteria in the natural environment (Street and Schaller 2016).

A third photoreceptor implicated in *Synechocystis* phototaxis is the BLUF protein PixD. *PixD* mutant cells show negative phototaxis under white and red light (Masuda and Ono 2004; Okajima *et al.*^2005^). Thus, PixD seems not to be specifically involved in a blue light response, although BLUF proteins are classically blue light photoreceptors. Unlike PixJ1 and UirS, PixD is not a membrane-integral protein, and its location in the cell is so far unclear. Under blue light, *pixD* mutants are non-motile like the wild type, but they become motile when the *pixD* mutation is combined with the *cph2* mutation. Under these conditions, the *pixD* mutant also showed negative phototaxis (Fiedler, Börner and Wilde 2005). PixD interacts with the CheY-PATAN response regulator PixE in a light-dependent manner. The crystal structure demonstrated that two asymmetric pentameric rings of PixD form a decamer with two monomeric PixE subunits binding to the surface of each ring (Yuan *et al.*^2006^; Ren *et al.*^2013^). This complex is stable in the dark, but dissociates upon blue light exposure into PixD dimers and PixE monomers (Yuan and Bauer 2008; Tanaka *et al.*^2011^). Because two PixD subunits should be photoactivated to induce disassembly of the complex, Tanaka *et al.* (2012) suggest that PixD might be a sensor of light intensity and that weak blue light is not able to release PixE from the complex. So far, it is not clear how PixE regulates directional sensing. In the *pixD* mutant, which shows negative phototaxis, PixE most probably is still synthesized, because it is the first gene in the putative operon comprising the transcriptional unit TU2063 (Kopf *et al.*^2014^) and the phenotype of a *pixDE* double mutant is distinct from the *pixD* single mutant (Sugimoto *et al.*^2017^). This suggests that free monomeric PixE changes the direction of movement by a so far unknown mechanism.

In summary, directional light sensing in *Synechocystis* is clearly complex. At least three photoreceptors (PixJ1, UirS and PixD) influence phototaxis, but it is not certain which, if any, of these photoreceptors are involved in directional light perception, since photoreceptors can also influence phototaxis in other ways (for example, by promoting the transcription of specific signal transduction components required for positive or negative phototaxis, or by providing post-translational signals that acclimate the system to different prevailing light conditions) (Fig. 4). PixJ1 is probably the best candidate for a directional light sensor, but it cannot be the sole directional light sensor, since *pixJ1* mutants still show negative phototaxis and must therefore be capable of directional light perception. Even further complexity is indicated by the involvement of Cph2, which provides light-dependent control of the activity rather than the direction of motility. Although we are a long way from fully understanding the system, the involvement of multiple photoreceptors (one of them also linked to an ethylene-sensing domain) suggests that *Synechocystis* must make complex decisions about its motility, influenced by multiple light and chemical signals.

### Light-controlled motility in filamentous cyanobacteria

True phototaxis responses are not confined to unicellular cyanobacteria. An extensive older literature on phototaxis in filamentous cyanobacteria (from the orders *Oscillatoriales* and *Nostocales*) was reviewed by Häder (1987). Photobehavior observed in filamentous cyanobacteria includes positive photokinesis which appears linked to the energy supply from the photosynthetic apparatus but also phototactic orientation. Filaments of *Phormidium* appear able to detect when they are migrating towards a light source, since motility reversals are suppressed under these conditions (Gabai 1985). This effect requires true directional light sensing since it can be observed under parallel illumination in which all parts of the filament are equally illuminated (Häder 1987). Motile *Anabaena* filaments are even able to steer themselves, with the front end of the filament turning either towards or away from the light, depending on light intensity (Nultsch, Schuchart and Höhl 1979). Experiments based on illuminating specific regions of the filament suggest that each individual cell is capable of detecting light direction (Nultsch and Wenderoth 1983). Thus, phototaxis in filamentous cyanobacteria seems to share most features in common with phototaxis in unicellular species such as *Synechocystis* (discussed above) perhaps with the added complication that intercellular communication within the filament could also influence motility. Action spectra of the phototactic orientation of several filamentous strains implied that photosynthetic pigments are involved in positive and negative phototaxis responses. However, experiments with the electron transport inhibitors DCMU and DBMIB suggested that the photosynthetic electron transport chain is not involved in phototactic orientation but only affects photokinesis (Häder 1987).

Recent progress in the study of phototaxis and motility in filamentous cyanobacteria includes the demonstration that hormogonia of *N. punctiforme* use a modified type IV pilus apparatus for their motility (Khayatan, Meeks and Risser 2015), providing a further point of similarity with the unicellular species (Wilde and Mullineaux 2015). Another major step forward is the identification of a photoreceptor for phototaxis in *N. punctiforme* hormogonia (Campbell *et al.*^2015^). The receptor protein PtxD (NpF2164) is a particularly complex multidomain cyanobacteriochrome containing seven GAF domains, at least six of which bind a bilin. In two of these domains, the bilin is linked to a second cysteine, thereby shifting the absorption peaks to the blue region. PtxD can undergo photoconversions between six different colors in total (Rockwell, Martin and Lagarias 2012). Hormogonia of a *N. punctiforme* mutant lacking PtxD retain their motility but lose the ability of the wild type for positive phototaxis, instead the mutant filaments move randomly under unidirectional illumination (Campbell *et al.*^2015^). The cyanobacteriochrome domains of PtxD are fused to a C-terminal MCP domain, and furthermore, the *ptx* gene cluster encodes homologs of CheY, CheW and CheA (Campbell *et al.*^2015^). This is strikingly reminiscent of the *tax1* gene cluster in *Synechocystis* (Bhaya, Takahashi and Grossman 2001), suggesting that onward signal transduction is similar to the *Synechocystis* cyanobacteriochrome PixJ1. It now seems very likely that phototaxis in unicellular and filamentous cyanobacteria work by similar mechanisms.

## CURRENT QUESTIONS

In this section, we briefly summarize some of the many open questions in the prokaryotic phototaxis, photobehavior and directional light perception.

### Localization and interactions of photoreceptors, signal transducers and the motility apparatus

There is no example of prokaryotic photobehavior that is fully understood in the sense that the complete signal transduction pathway from the photoreceptor to the motility apparatus is known. Even in the well-characterized case of *H. salinarum*, the final switch that controls the direction of rotation of the archaellum is not understood. In cyanobacteria, a plethora of photoreceptors and signal transducers that influence phototaxis are known. However, it remains uncertain which of these components are involved in directional light perception, and knowledge of the signal transduction pathway leading to control of the activity of type IV pili remains incomplete. We feel that two lines of further investigation are needed. First, we need to know the subcellular localization of photoreceptors and signal transducers, ideally with dynamic information on changes in localization during motility switches. This information could in principle come from a combination of fluorescent protein tagging and fluorescence microscopy, although the low copy number of many signal transduction proteins may cause problems with this approach in cyanobacteria, which have a relatively high background autofluorescence. Secondly, we need to know much more about intermolecular interactions. For example, which, if any, response regulators directly interact with the type IV pilus motors in cyanobacteria? This information could come from a range of techniques: e.g. *in vivo* FRET, affinity pull-downs and two-hybrid systems.

### Adaptation and dynamic range

A common feature of photoperception systems in all domains of life is the presence of adaptation that tunes photoperception and signal transduction to the prevailing light intensity. Adaptation effectively increases the dynamic range of the system, allowing it to respond over a much wider range of prevailing light intensities. Such adaptation mechanisms are likely to be a feature of prokaryotic photobehavioral responses, but we know very little about how they might operate. The methylation of *R. sphaeroides* (Kort *et al.*^2000^) and *H. salinarum* (Perazzona and Spudich 1999) MCPs are rare examples of prokaryotic light adaptation responses that are at least partially understood. In the cyanobacterium *Synechocystis*, light focusing by the cells induces a roughly 4-fold difference in light intensity between the front and back of the cell (Schuergers *et al.*^2016^). However, the range of light intensities over which *Synechocystis* phototaxis operates appears much greater than this (Ng, Grossman and Bhaya 2003), which indicates the need for a light adaptation system. Some of the numerous *Synechocystis* photoreceptors and signal transducers that are implicated in phototaxis (Table 2) may be involved in adaptation to the prevailing light conditions rather than directional light perception or switching between positive and negative phototaxis, but the details remain to be unraveled.

### Integrating multiple signals for complex decisions

As discussed above, the choice of optimal light conditions for photoautotrophic growth could be extremely complex, involving assessment of nutrient and gas supply and the presence of competitors, symbiotic partners and predators as well as a simple assessment of light quality and intensity. For good reason, we tend to minimize these complicating factors in laboratory experiments where we seek reproducible results and answers to simple questions. Nevertheless, there are hints in several prokaryotes for the complexity of information processing that may go into motility decisions in the real world. In purple photosynthetic bacteria, there is likely cross-talk between light signals, the metabolic status of the cell and oxygen levels (Armitage 1997). In cyanobacteria, the situation appears even more complex. *Synechocystis* is equipped with multiple photoreceptors and makes sophisticated choices about its direction of motility in complex light regimes (Chau, Bhaya and Huang 2017). Furthermore, ethylene has been shown to influence phototaxis via its interaction with the UirS UV-A photoreceptor (Lacey and Binder 2016). The presence of the *tax2* and *tax3* operons, whose products likely influence motility in response to uncharacterized chemical or mechanical signals, hints at further complexity in the control of *Synechocystis* motility. The nature of complex information processing in these tiny single cells will be a fascinating topic for future study.

### Photobehavior and directional light perception in non-phototrophic prokaryotes

Many non-phototrophic prokaryotes possess photoreceptors, and light signals have been shown to influence a number of physiological processes in non-phototrophs, including development and virulence (Purcell and Crosson 2008; Bonomi *et al.*^2016^). We are not aware of any instance in which it has been shown that a non-phototrophic prokaryote employs directional light sensing, as has been shown for the cyanobacterium *Synechocystis* (Schuergers *et al.*^2016^). However, since the basis of directional light sensing in *Synechocystis* is microoptical lensing rather than shading (Schuergers *et al.*^2016^), a high cellular pigment content is not a requirement for directional light perception. Our own preliminary investigations suggest that microoptic lensing and waveguiding effects are possible in a range of non-phototrophic microbes, suggesting that directional light perception could potentially be widespread in non-phototrophic prokaryotes. To take one example, *Agrobacterium tumefaciens* has a pair of bacteriophytochrome photoreceptors (Karniol and Vierstra 2003) and a developmental pathway involving the generation of a substrate attachment site at one pole of the cell (Heindl *et al.*^2014^). Does directional light sensing play a role in determining which cell pole becomes the attachment site? Studies of subcellular localization of photoreceptors would provide a first clue to their possible involvement in directional light sensing. Such photoreceptors should be located around the periphery of spherical cells, or at the cell poles in rod-shaped cells.

### What is the role of photobehavior in the real world?

Nearly all our knowledge of prokaryotic photobehavior comes from laboratory studies of cells in relatively simple situations. Such studies give obvious clues to possible advantages of photobehavior in the natural environment, but they do not directly reveal the circumstances in which photobehavior might be selectively advantageous. A rare example of the observation of prokaryotic photobehavior in the natural environment comes from studies of the diel migration of the filamentous cyanobacterium *Oscillatoria* sp. within mat communities, where it is clear that photosensing has a major influence on movement, although it is not the only factor (Richardson and Castenholz 1987; Garcia-Pichel, Mechling and Castenholz 1994). Cyanobacterial species migrate vertically within biofilms over distances of the order of 1 mm during diurnal cycles, migrating downwards during the day and upwards during the night (Garcia-Pichel, Mechling and Castenholz 1994). The complex photobehavior of unicellular species such as *Synechocystis* must be important in similar contexts, but this remains to be investigated. Circadian rhythms and diurnal cycles greatly influence the physiology of cyanobacteria (Angermayr *et al.*^2016^) and could make yet another input into motility decisions in unicellular cyanobacteria. To our knowledge, this has not yet been tested. Someday we hope to understand how cyanobacteria process complex information from multiple environmental cues to arrive at motility decisions, and how this behavior is relevant to their survival in the natural environment.
